# Maternal Oxycodone Treatment Results in Neurobehavioral Disruptions in Mice Offspring

**DOI:** 10.1523/ENEURO.0150-21.2021

**Published:** 2021-08-04

**Authors:** Rachel E. Martin, Madison T. Green, Jessica A. Kinkade, Robert R. Schmidt, Tess E. Willemse, A. Katrin Schenk, Jiude Mao, Cheryl S. Rosenfeld

**Affiliations:** 1 Christopher S Bond Life Sciences Center, University of Missouri, Columbia, MO 65211; 2 Biomedical Sciences, University of Missouri, Columbia, MO 65211; 3 Physics, Randolph College, Lynchburg, VA 24503; 4 MU Institute for Data Science and Informatics, University of Missouri, Columbia, MO 65211; 5 Thompson Center for Autism and Neurobehavioral Disorders, University of Missouri, Columbia, MO 65211; 6 Genetics Area Program, University of Missouri, Columbia, MO 65211

**Keywords:** analgesic, brain, DOHaD, drug abuse, gene expression, opioid

## Abstract

Opioid drugs are increasingly being prescribed to pregnant women. Such compounds can also bind and activate opioid receptors in the fetal brain, which could lead to long-term brain and behavioral disruptions. We hypothesized that maternal treatment with oxycodone (OXY), the primary opioid at the center of the current crisis, leads to later neurobehavioral disorders and gene expression changes in the hypothalamus and hippocampus of resulting offspring. Female mice were treated daily with 5 mg OXY/kg or saline solution (control; CTL) for two weeks before breeding and then throughout gestation. Male and female offspring from both groups were tested with a battery of behavioral and metabolic tests to measure cognition, exploratory-like, anxiety-like, voluntary physical activity, and socio-communication behaviors. qPCR analyses were performed for candidate gene expression patterns in the hypothalamus and hippocampus of OXY and CTL derived offspring. Developmental exposure to OXY caused socio-communication changes that persisted from weaning through adulthood. Such offspring also showed cognitive impairments, reduced voluntary physical activity, and weighed more than CTL counterparts. In the hippocampus, prenatal exposure to OXY caused sex-dependent differences in expression of genes encoding opioid receptors and those involved in serotonin signaling. OXY exposure induced changes in neuropeptide hormone expression and the epigenetic modulator, *Dnmt3a*, in the hypothalamus, which could result in epigenetic changes in this brain region. The findings suggest cause for concern that consumption of OXY by pregnant mothers may result in permanent neurobehavioral changes in their offspring. Further work is needed to determine the potential underpinning epigenetic mechanisms.

## Significance Statement

Opioids continue to be one of the most commonly prescribed analgesics, but they are highly addictive. Opioid use disorder (OUD) is a problem in women of childbearing age. This study used a mouse model to examine whether maternal treatment with oxycodone (OXY) during pregnancy would lead to long-term neurobehavioral alterations in her offspring. Results from this study indicate that developmental exposure to OXY results in longstanding behavioral impairments and gene expression changes in the brain of mice offspring. Findings from this study thus suggest potential cause for concern that similar impairments may occur in infants born to mothers with OUD.

## Introduction

Opioids continue to be one of the most commonly prescribed analgesics, but they are highly addictive. Consequently, prescription opioid pain relievers were abused in 2016 alone by ∼4% of the United States population (Reinhart et al., 2018). Opioid abuse is considered one of the leading non-communicable, public health diseases and economic challenges in the United States (Reinhart et al., 2018). Oxycodone (OXY; OxyContin) is one of the primary opioid drugs persistently prescribed and abused. Opioid use disorder (OUD is a problem in women of childbearing age. A Substance Abuse and Mental Health Services Administration (SAMHSA) report showed female treatment admissions for opioid pain relievers greatly outnumbered male admissions in all age categories (Substance Abuse and Mental Health Services Administration, 2015).

OXY and other opioids are prescribed for pregnant women, which has resulted in OUD during pregnancy affecting ∼5.6 per 1000 live births (Patrick et al., 2012). More than 85% of pregnancies with women with OUD are left untreated (Heil et al., 2011). Pregnant women with OUD show delayed and reduced rates of prenatal care compared with those without substance use disorders (Clemans-Cope et al., 2019). Infants prenatally exposed to opioids are at risk for neonatal abstinence syndrome (NAS or neonatal opioid withdrawal syndrome; Jones et al., 2019). Birth outcomes associated with maternal OUD include poor fetal growth, potential premature birth, low birthweight, and possible congenital defects (Yazdy et al., 2015; Centers for Disease Control and Prevention, 2019). One study revealed that such infants had elevated neonatal intensive care unit (NICU) admission rates and required longer hospitalization periods (Clemans-Cope et al., 2019). Thus, overall health care costs for women with OUD and their infants is substantially greater than those without this disease (Clemans-Cope et al., 2019). Those infants born to mothers with OUD may not show health effects early on, but may be vulnerable to later diseases because of developmental origin of health and disease (DOHaD) effects (Grandjean et al., 2015; Rosenfeld, 2015). Endogenous opioids are subject to homeostatic mechanisms and regulate key events during fetal brain development (Mohamed et al., 2020). As such, developmental exposure to opioid drugs can also affect offspring brain development and risk for later neurobehavioral disorders (Vathy, 1995; Sanchez et al., 2008; McPherson et al., 2015; Ross et al., 2015; Akbarabadi et al., 2018; Goldfarb et al., 2020; Jantzie et al., 2020; Mohamed et al., 2020).

Maternal exposure to morphine impairs brain development and weight of the whole brain and cerebellum in rats (Zagon and McLaughlin, 1977). Prenatal exposure to opiates in a rodent model appears to lead to long-lasting alterations in the neuroendocrine control systems, especially in the hypothalamic-pituitary gland axis (Litto et al., 1983). Learning and memory is one of the primary behavioral domains that has been examined in rodent offspring developmentally exposed to OXY or other opioid drugs. Male Sprague Dawley rats developmentally exposed to OXY have impairments in spatial learning and memory when tested in the T-maze and Morris water maze (Davis et al., 2010). Spatial memory of male but not female Wistar rats and grand-offspring is compromised in those derived from dams treated with morphine (Odegaard et al., 2020). Correspondingly, *Mecp2* and *Hdac2* were significantly upregulated in the hippocampus of these male groups. Rat offspring derived from female and male parents treated with morphine exhibit memory deficiency that is presumably because of suppression of long-term potentiation in the hippocampus (Sarkaki et al., 2008). Additionally, anxiogenic behaviors have been reported in rat offspring developmentally exposed to morphine (Li et al., 2014).

Based on these previous studies, we hypothesized that developmental exposure of mice to OXY would lead to longstanding effects on the above and other behavioral domains, including exploratory-like, anxiety-like, and socio-communication behaviors. Additionally, we sought to determine whether such behavioral changes correlated with gene expression changes, especially those encoding opioid receptors and genes associated with other neurotransmitter-reward signaling pathways, in particular serotonin (5-HT) and dopamine, in the hypothalamus and hippocampus. To test this hypothesis, CF1 female mice were exposed to OXY or saline control (CTL) two weeks before breeding (periconception period) and then throughout gestation. Male and female offspring behaviors were tested at juvenile period and adulthood. Quantitative (q)PCR was used to examine candidate gene, including various opioid receptors, expression in the hypothalamus and hippocampus, two brain regions that regulate these key behaviors.

## Materials and Methods

### Animals and treatments

Current animal experiments were approved by our Institutional Animal Care and Use Committee (ACUC, protocol #9590). All studies conformed to the National Institutes of Health Guidelines for the Care and Use of Laboratory Animals. Seven-week-old male and female CF1 mice were ordered from Envigo, and females were habituated to the animal facility for one week before being placed on one of two treatments. Mice were maintained on a 12/12 h light/dark cycle with the lights turning on at 7 A.M. and shutting off at 7 P.M. The average room temperature is 70 °F, and the humidity range is between 30–70%. Female mice were randomly assigned to be in the OXY (catalog #O1378; Sigma Chemical) or saline CTL groups. At eight weeks of age, the OXY group received 5 mg OXY/kg body weight in 0.9% saline with an average volume of 0.1 ml injected intraperitoneally between in the morning daily for two weeks before breeding and then throughout gestation. During this time, the CTL group received comparable intraperitoneal injection volumes of 0.9% saline. Females were weighed weekly throughout the course of the experiment, and the dose of OXY was adjusted to continue to provide a dose of 5 mg/kg. This dose and route of administration (intraperitoneal) was used based on past findings that showed such concentrations mimic those achieved in humans with OUD (Liu et al., 2005; Zhang et al., 2009; Szumlinski et al., 2020). No ill. effects were noted in mice treated with OXY or saline CTL intraperitoneal injections. No differences in appetite or weight gain were noted for females in the OXY or saline CTL group. The treatments commenced two weeks before breeding to include the periconceptional period, as this may be important in preimplantation embryonic development (Mao et al., 2010, 2020). Animals were provided food and water *ad libitum* and fed an AIN93G phytoestrogen-free diet (Envigo) to reduce any exogenous estrogen exposure.

### Breedings

**Figure 1.**
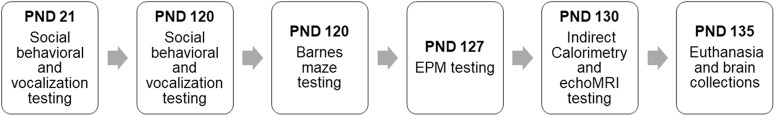
Timeline for the behavioral and metabolic studies. PND = postnatal day.

After two weeks of being treated daily with OXY or CTL solutions, females were paired with potential CF1 breeder males and examined the next morning for a vaginal plug. The day a vaginal plug was observed was considered embryonic day (E)0.5. If no vaginal plug was observed in the morning, males were placed in separate cages and re-paired that evening with females. Female mice were maintained on their respective treatments until parturition. No differences in fertilization rates or pregnancy success rate were noted between the two maternal groups. One male and one female offspring from each litter (*n* = 13 male and 13 females for CTL and 10 male and 10 female mice for OXY group) were randomly chosen to undergo behavioral and metabolic testing. The same male and female offspring from each litter were used for all of the behavioral and metabolic assessments and gene expression studies. The timeline of the studies is shown in Figure 1.

### Social behavior testing

At postnatal day (PND)21 and PND120, male and female offspring underwent Crawley’s sociability and preference for social novelty three-chambered test, as detailed previously (Moy et al., 2004; Marshall et al., 2019). This was done to examine for potential social deficits because of developmental exposure to OXY. Tests were conducted in the morning. The apparatus allows the test mouse to move freely between the three chambers by using two openings. The left and right chambers contained wire mesh cups that held stranger (novel) mice that were similar in age, sex, and parental diet to the test animal. The stranger mice had different parental lineages relative to the test mouse. Test and stranger individuals were habituated to the testing room for 30 min before the start of the experiments. The test consisted of three trials. In the first trial, the test mouse was placed in the center chamber and could roam freely within the chamber for 5 min. This allowed the mouse to acclimate to the maze. No strangers were placed in their wire cups in the first trial. Between trial 1 (T1) and T2, novel strangers were acclimated to the wire mesh cups for 5 min. In T2, stranger 1 was placed under the wire cup on the left chamber while the test individual was placed in the center of the maze. In T3, the test mouse was placed in the center chamber and stranger 1 and 2 individuals were placed under the cup in the left and the right chambers. T2 and T3 lasted for 10-min duration to provide sufficient time to observe novel behaviors while avoiding habituation. Trials were video recorded using a Logitech Carl Zeiss Tessar HD 1080P camera mounted to a Joby Gorilla Pod Original Tripod (Daymen US Inc.). Video data were analyzed using Observer software version 11.5 (Noldus). Behaviors such as rearing, grooming, and nose-to-nose contacts with the stranger mice (in T2 and T3) were determined, along with the location of the mouse within the three-chambered apparatus.

### Ultrasonic and audible vocalizations

Following T3 of social testing at postnatal day (PND)30 and 120 d of age, ultrasonic and audible vocalizations produced in isolation were measured, as described previously (Johnson et al., 2018b). The mouse was placed in a clean polypropylene box, which was then placed in chamber lined with 2 inches of acoustic foam paneling (Soundproof Cow). The box contained a light source (20-watt LED puck lights, Intertek) and an Avisoft Bioacoustics CM16/CMPA40-5V microphone (Glienicke) that was 33 cm from the floor of the box. A microphone was attached to National Instruments USB 6351 data collection board, which was connected to a Dell OptiPlex 7010 (Dell Incorporated). Vocalizations were recorded for 5 min. Recordings consisted of audible calls (syllables) and ultrasonic vocalizations (USVs), which are above 20 kHz and outside of the hearing range for humans. After each test, the box was cleaned with 70% ethanol, which also removed previous odor cues. Data were collected using a code accepted for rodent vocalizations and MATLAB 2015a.Ink version 8.5.0.197613 (R2015a) software (The MathWorks). Categories evaluated included number of syllables, syllable duration, syllable median frequency, average syllable power, and power percent above and below 20 kHz, as detailed previously (Johnson et al., 2018b).

### Barnes maze testing

At PND120 (following social and vocalization studies), test mice underwent Barnes maze testing to assess spatial learning and memory, as described previously (Jašarević et al., 2011, 2013; Rosenfeld, 2013; Rosenfeld and Ferguson, 2013; Williams et al., 2013). Briefly, the Barnes apparatus is a circular platform with 12 holes along the perimeter. Four intra-maze visual cues were placed above holes 3, 6, 9, and 12. A bright light was positioned above the maze, as a non-noxious stimulus, to encourage the mice to seek the escape hole. The size of the maze and illumination used for the lights is the same as reported previously (Jašarević et al., 2011, 2013; Rosenfeld, 2013; Williams et al., 2013). Mice were randomly assigned to an escape hole at the beginning of the test, and the assigned escape hole was constant throughout the 5-d testing period. Mice were placed in the testing room for 30 min before testing to allow for acclimation. The mouse was placed under a clean polypropylene box in the center of the maze, the box was removed, and timer started. The test concluded once the test mouse entered the escape hole or when 5 min had elapsed. If the test mouse did not enter escape hole on first day of testing, it was gently guided to the escape hole. After a 30-min interval, a second trial was performed. The maze platform, polypropylene box, and escape hole were cleaned with 70% ethanol between each trial and each mouse. This procedure also removed odor cues that could affect the performance of subsequent individuals. The tests were recorded with a Sony Handycam HDR-CX440. Videos were analyzed using ANY-maze software version 6.2 (Stoelting). The program tracked the movement of the mouse and analyzed various behaviors, including latency to find the correct escape hole, number of entries into correct and incorrect quadrants, total distance traveled, and velocity.

### Elevated plus maze (EPM)

At PND127, individuals were then tested in the EPM, which measures anxiety-like behavior, exploratory, and repetitive behaviors, as described previously (Fountain et al., 2008; Jašarević et al., 2011, 2013; Williams et al., 2013). The apparatus consists of two open and two closed arms connected at the center and perpendicular to each other. The arms were raised 100 cm off the floor. The closed arms had walls that enclosed the space from the surroundings. Test individuals were acclimated to the testing room for 30 min before testing. The test began when the mouse was placed at the center and concluded after 5 min. The apparatus was cleaned with 70% ethanol between each trial, which also removed previous odor cues. The test was recorded with a Sony Handycam HDR-CX440 and then analyzed by using the Observer software version 11.5 (Noldus). Frequency and duration of various behaviors were analyzed including grooming, rearing, head dipping, time spent in center, open arms, and closed arms.

### Indirect calorimetry testing

After completion of the behavioral trials, male and female offspring from both groups underwent indirect calorimetric testing for 3 d, where they were tested in the Promethion continuous measurement indirect calorimetry system (Sable Systems International), as we have described previously (Johnson et al., 2015, 2017b; Butler et al., 2020). Data were divided into three 12-h light cycles and three 12-h dark cycles. Categories evaluated include general activity level, energy expenditure, respiratory quotient, body mass, and food and water intake.

### EchoMRI

After indirect calorimetry testing, mice were tested in the EchoMRI-1100 (EchoMRI LLC) to analyze body composition, as described previously (Johnson et al., 2015, 2017b; Butler et al., 2020). Parameters measured include lean tissue, fat tissue, and water content.

### Hypothalamic and hippocampal punches and RNA isolation

Upon completion of all assessments, mice were humanely killed, and brains were removed from the cranium and rapidly frozen on dry ice. The frozen brains were stored at −80°C until further processing. The hypothalamic and hippocampal regions were micro-punched on dry ice, as done previously (Johnson et al., 2017a, 2018a; Butler et al., 2020). A Harris Micro-Punch 1 mm in diameter and 1 mm in depth (catalog #15091, Ted Pella) was used to obtain two bilateral punches that spanned the rostral to caudal medial regions of this brain region. RNA and miRs/small RNAs were isolated by using the AllPrep DNA/RNA/miRNA Universal kit (catalog #80224; QIAGEN). After RNA isolation, RNA was quantified by spectrophotometrical analysis (Nanodrop 2000, ThermoFisher Scientific). The results were further confirmed by analyzing the RNA on the Fragment Analyzer (Agilent). Not all of the brain samples were used for these analyses as we sought to save approximately half the brain samples for other analyses, in particular epigenetic studies. Based on our previous studies and power analyses, five to seven samples per group should provide sufficient replicates to detect potential differences between groups.

### Quantitative q(PCR) validation

Several candidate genes, as listed in Table 1, were analyzed by qPCR analysis. Total RNA, which had been treated with DNase to remove any genomic DNA contamination, was reverse transcribed into cDNA using the QuantiTect Reverse Transcription kit (catalog #205310, QIAGEN). The qPCR procedure was performed on The Bio-Rad CFX Connect Real-Time PCR Detection System (Bio-Rad). Primers were designed by using NCBI Primer-Blast online (http://www.ncbi.nlm.nih.gov/tools/primer-blast/) and purchased from IDT technologies. Primer sequences and efficiencies for the genes examined are listed in Table 1. The Bio-Rad SYBR Green Master Mix (catalog #1725121) was used according to the manufacture’s protocol. The cycling conditions for qPCR were (1) 95°C for 5 min for polymerase activation (2) 40 cycles of: denaturation for 15 s at 95°C followed by annealing and extension for 30 s at 56°C, and (3) dissociation melt curve analysis and denaturation at 95°C, complete annealing at 56°C, followed by a gradual increase in temperature up to 95°C. Both *B2m* and *Rpl7* genes were used as internal reference controls. Two internal reference controls that showed strong similarity in Ct results were included to increase the strength and confidence in the comparisons.

**Table 1:** Primers used for hippocampus and hypothalamus qPCR in male and female CF1 mice offspring

Gene	Forward primer (5′ to 3′)	Reverse primer (5′ to 3′)	Efficiency
*Avp*	ATCTGCTGCAGCGACGAGAG	TGTACCAGCCTTAGCAGCAG	90.3
*B2m1*	CCTTCAGCAAGGACTGGTCT	TTGATCACATGTCTCGATCCCAG	100.3
*Bdnf*	CGGAGAGCAGAGTCCATTCAG	CTCACCTGGTGGAACTTCTTTG	90.7
*Cnrip1*	AACACTGCAGGTCGAGAACATT	TTGCCACACTGTCTCGAAGG	91.3
*DAT*	CTGGAAGATCTGCCCTGTCC	CAGCACACCACGCTCAAAAT	103.9
*Dnmt3a*	GATGAGCCTGAGTATGAGGATGG	CAAGACACAATTCGGCCTGG	103.6
*Dnmt3b*	CTGTCCGAACCCGACATAGC	CCGGAAACTCCACAGGGTA	96.4
*Drd1*	TTGAGTCCAGGGGTTTTGGG	GGGCCTCTTCCTGGTCAATC	99.0
*Drd2*	TGAACAGGCGGAGAATGGATG	GTTTTGCCATTGGGCATGGT	90.0
*Esr1*	CTTGAACCAGCAGGGTGGC	AGGCTTTGGTGTGAAGGGTC	100.2
*Esr2*	ACGAAGAGTGCTGTCCCAAG	GCCAAGGGGTACATACTGGAG	112.0
*Gnrh*	CAACTGTGCTCACCAGCGG	TGAGGATCATGTCCACTCTGTTT	110.0
*Htr2a*	CACCGACATGCCTCTCCATT	GGCCACCGGTACCCATACAG	90.9
*Htr7*	GGGACCTGAGGACCACCTAT	CAGTGGTCACAGTTTTGTAGCA	98.0
*Kiss1*	AGACTGTAGACCTGCCCCTT	GACTGCTGGCCTGTGGAT	106.7
*Lepr*	CAGAATGACGCAGGGCTGTA	GCTCAAATGTTTCAGGCTTTTGGA	92.7
*Oprd1*	CCGTGGCCTCCGTTTTCC	CAATTTGGTGTACCGGACGA	95.0
*Oprk1*	AGTGCCACCTTCTCGCTTT	GGTCTTCATCTTCGTGTATCGGA	99.3
*Oprl1*	TCTTCGGAGCAGGAGCTAGG	GCCACTCAGTACAGTTCCTCC	105.4
*Oprm1*	CATGGCCCTCTATTCTATCGTGT	CCACGTTCCCATCAGGTAGTT	99.4
*Oxtr*	GTCTGGTCAAATACTTGCAGGT	CGCGCAGCGAGAAAATGTG	97.6
*Rpl7*	AGCTGGCCTTTGTCATCAGAA	GACGAAGGAGCTGCAGAACCT	96.1
*Slc6a4*	CTCCGCAGTTCCCAGTACAAG	CACGGCATAGCCAATGACAGA	108.5

### Statistical analyses

Litter size at birth was analyzed by using PROC general linear model (GLM) procedure of SAS with maternal OXY treatment as the main factor. Pup sex ratio data were analyzed by using the generalized linear model of the SAS version 9.4 (PROC GENMOD procedure, SAS Institute). The data were distributed as a binomial and transformed by means of a logit-link function. The χ^2^ test was used to test deviation from a 1:1 ratio (i.e., a value of 0.5 for the fraction of male fetuses), as well as for differences in sex ratio between groups. The antilog of the logit and the antilog of the differences between logit estimates produced the odds and odds ratio, respectively.

Behavioral and metabolic data were analyzed by using SAS version 9.4 Software (SAS Institute). A split plot in space and time (an ANOVA-based method) was used to analyze the data, as detailed by (Steel, 1996) . The main plot consisted of maternal treatment, and the subplot was F1 pup sex and maternal treatment × F1 pup sex.

Behavioral assessments listed above were first recorded with a camera, with the manners noted above, timed using a stopwatch, and recorded. All dependent variables including social behaviors, ultrasonic and audible vocalizations, and EPM tests were analyzed by using the GLM procedure of SAS. Sources of variation considered were maternal treatment, offspring sex, and interaction between maternal treatment and offspring sex. Treatment effects were determined with dam as the error term (experimental unit). Differences between OXY-exposed and CTL groups were determined by Fisher’s protected LSD. The LSD was only calculated if the overall *F* test was significant.

Dependent variables assessed in the Barnes maze, including distance traveled and velocity were analyzed as a split plot in space and time (Steel, 1996). The linear statistical model contained the fixed effects of maternal treatment, sex, day, and all possible interactions with treatment, offspring sex, and day. To determine whether there were litter effects, source (dam) within treatment was used as the denominator of *F* for maternal treatment, source within day × sex was used as the denominator for sex and interaction of maternal treatment × offspring sex, source within day was used as the denominator of *F* for day and the remaining interaction used the residual mean square as the denominator of *F*.

Latency data for the Barnes and reverse Barnes maze testing were further analyzed by using the PROC PHREG and Proportional Hazard Ratio functions in the SAS. These analyses adjust for right-censoring (defined here as not locating the escape hole within the allotted time of 300 s) while still accommodating the study design of 300 s/trial. Data are reported as a hazard ratio that signifies the odds of a subject in a treatment group locating the correct escape hole compared with the other groups tested. A significant result indicates the odds are not 1:1. A result >1 indicates the test group was more likely to locate the correct escape hole than all other groups tested. A result <1 indicates that the treatment group is less likely to locate the correct escape hole compared with the other study groups. The litter was used as the denominator of *F* for the effects of maternal treatment, offspring sex and test day, and potential interactions between maternal treatment, offspring sex and test day. Latency data are reported as the mean and 95% lower and upper confidence limits.

Indirect calorimetric testing data were analyzed as a repeated measurement analysis in which the main plot contained the effects of the maternal treatment and offspring sex. The denominator of *F* for the main plot was litter within maternal treatment and offspring sex. The subplot contained the time series of both day and cycle. The day and cycle were factorial arranged in which the cycle contained two cycles (dark and light) and day contained the 2 d in which animals were measured in this unit. The subplot effect of day and cycle and day × cycle and the interactions of day and cycle with the main plot effect were tested using litter within maternal treatment, offspring sex, day, and cycle as the denominator of *F*.

EchoMRI data were analyzed as a complete randomized design in which treatments were arranged as a two by two factorial (two maternal groups and two offspring sexes). Dam within maternal treatment was used as error term to determine maternal treatment effects. If the overall *F* was significant, then differences were determined using Fisher’s protected LSD. All data are presented as actual means and SEM. Gene expression data, as determined by qPCR analyses, were normalized by using combined average dCt values of the two housekeeping genes: *B2m* and *Rpl7* and then analyzed based on treatment × sex interactions with the PROC GLM procedure of SAS. If the overall *F* or interaction between treatment and sex was significant, then differences were determined using Fisher’s protected LSD. Graphs are based though on 2^-ΔΔCt^ values relative to CTL values for each sex with the mean value for these CTL groups set to 1 for graphing purposes. For all data, a *p* ≤ 0.05 was considered significant. All data are presented as mean ± SEM. Individual datapoints are also included in all graphs except for latency in the Barnes maze as these data were analyzed by using a hazard ratio approach to account for those mice that did not solve the maze in the allotted 600 s.

### Integrative correlation analyses

We used the mixOmics (v6.1.1) R package (Rohart et al., 2017) to correlate the behavioral, metabolic, and qPCR results. We conducted sparse discriminant analysis with partial least square regression with function *block.splsda*. The circos plot was generated by using the *circosPlot* function with correlations calculated by the method described by González et al. (2012). A correlation coefficient ≥0.75 was used as the cutoff.

## Results

### Litter results

Average litter size was 10.2 ± 1.0 and 8.5 ± 1.1 for the CTL and OXY groups, respectively, which was not significantly different. Male sex ratio was 54.4 ± 4.6% and 53.0 ± 6.0% for the CTL and OXY groups, respectively. These results did not differ between the two groups or from the expected ratio of 50:50.

### Socio-communication behaviors

**Figure 2.**
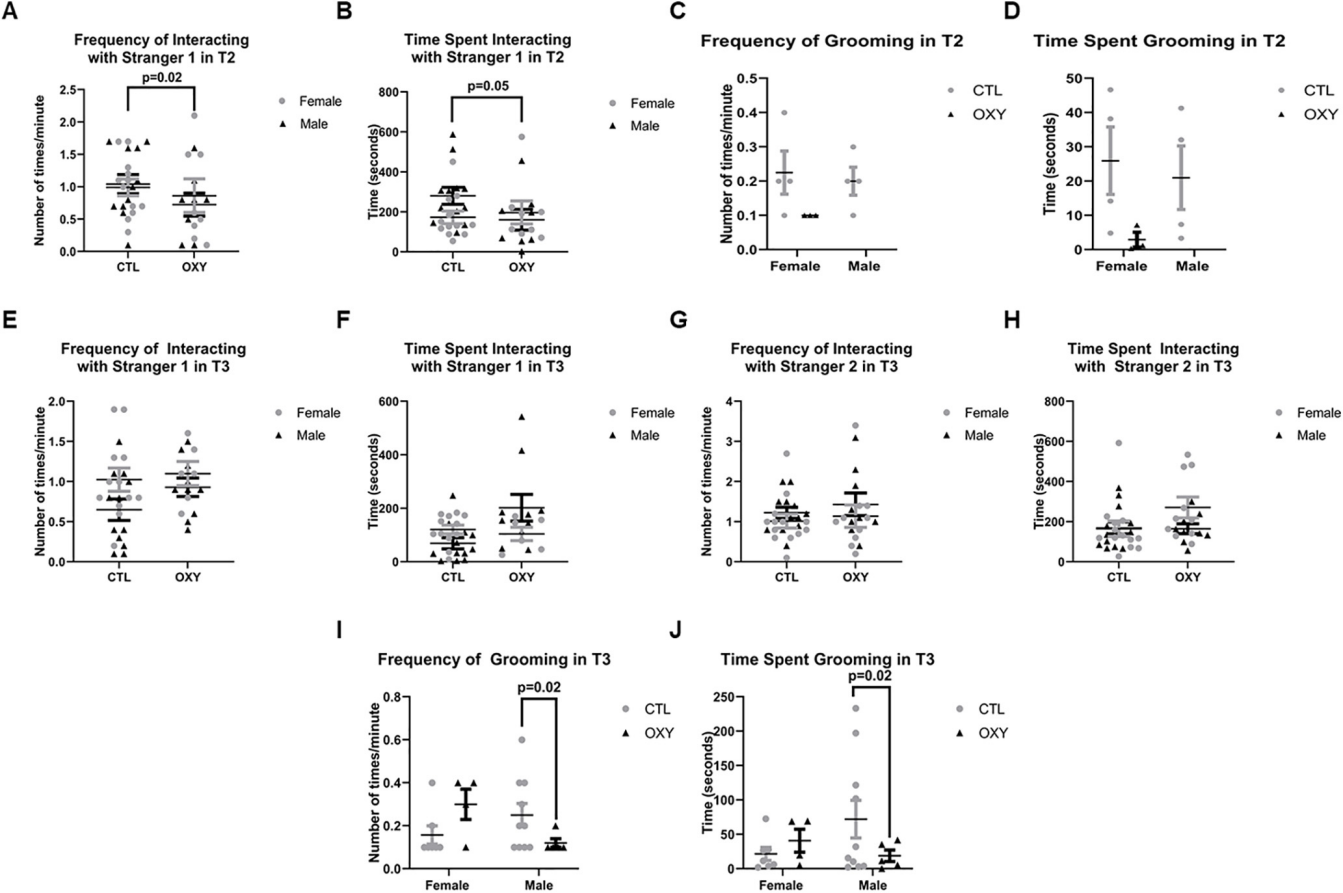
Social behavior results at PND21. ***A***, Frequency of interacting with stranger 1 in T2. ***B***, Time spent interacting with stranger 1 in T2. ***C***, Frequency of grooming in T2. ***D***, Time spent grooming in T2. ***E***, Frequency of interacting with stranger 1 in T3. ***F***, Time spent interacting with stranger 1 in T3. ***G***, Frequency of interacting with stranger 2 in T3. ***H***, Time spent interacting with stranger 2 in T3. ***I***, Frequency of grooming in T3. ***J***, Time spent grooming in T3. The *F* test was used for data analysis. Treatment, sex, and treatment by sex interaction were included in the model. Treatment effects were analyzed with dam nested under treatment and used as error term. There was significant (*p* < 0.05) interaction between treatment and sex in frequency and time spent in grooming in T3. Significant differences are indicated in the figures. Error bars represent SEM. Number of replicates tested = 13 female and 13 male mice for CTL group, 10 female and 10 male mice for OXY group. Datapoints for all replicates who engaged in the various behaviors are shown. For grooming in T2, many of the animals in both groups did not exhibit this behavior.

**Figure 3.**
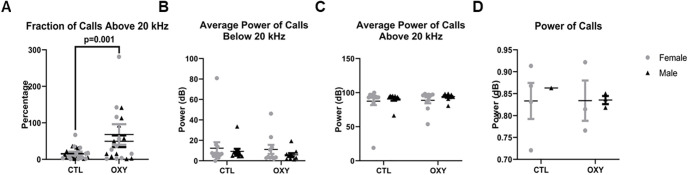
Audible and ultrasonic communications at PND21. ***A***, Fraction of calls in the ultrasonic range (above 20 kHz). ***B***, Average power of calls below 20 kHz. ***C***, Average power of calls above 20 kHz. ***D***, Power of calls. The *F* test was used for data analysis. Treatment, sex, and treatment by sex interaction were included in the model. Treatment effects were analyzed with dam nested under treatment and used as error term. There was significant (*p* < 0.01) treatment effect on fraction of calls above 20 kHz as indicated in ***A***. Error bars represent SEM. Number of replicates tested = 13 female and 13 male mice for CTL group, 10 female and 10 male mice for OXY group. Datapoints for all replicates who engaged in the various behaviors are shown. However, many of the animals in both groups did not exhibit any calls during the trial period.

At PND21, OXY-exposed offspring showed reduced frequency (df = 1, *F* = 6.5, *p* = 0.02) and duration (df = 1, *F* = 4.3, *p* = 0.05) interacting with a stranger mouse in T2 (Fig. 2). However, no differences were observed in interacting with stranger 1 or 2 mouse for T3 (Fig. 3). While no differences in grooming were noted during T2, during T3, males exposed to OXY had reduced incidences and durations of grooming themselves relative to CTL males (Fig. 3). It should be noted that many of the animals in both groups did not groom during T2.

Measurements of audible and USVs at PND21 revealed that OXY-exposed offspring were more likely (df = 1, *F* = 17.8, *p* = 0.001) to communicate in the ultrasonic range (>20 kHz; Fig. 3). No differences, however, were noted in power of calls below or above 20 kHz or average power of calls. This could also be explained though by the fact that several of the animals in both groups did not exhibit any calls at this age during the trial period.

**Figure 4.**
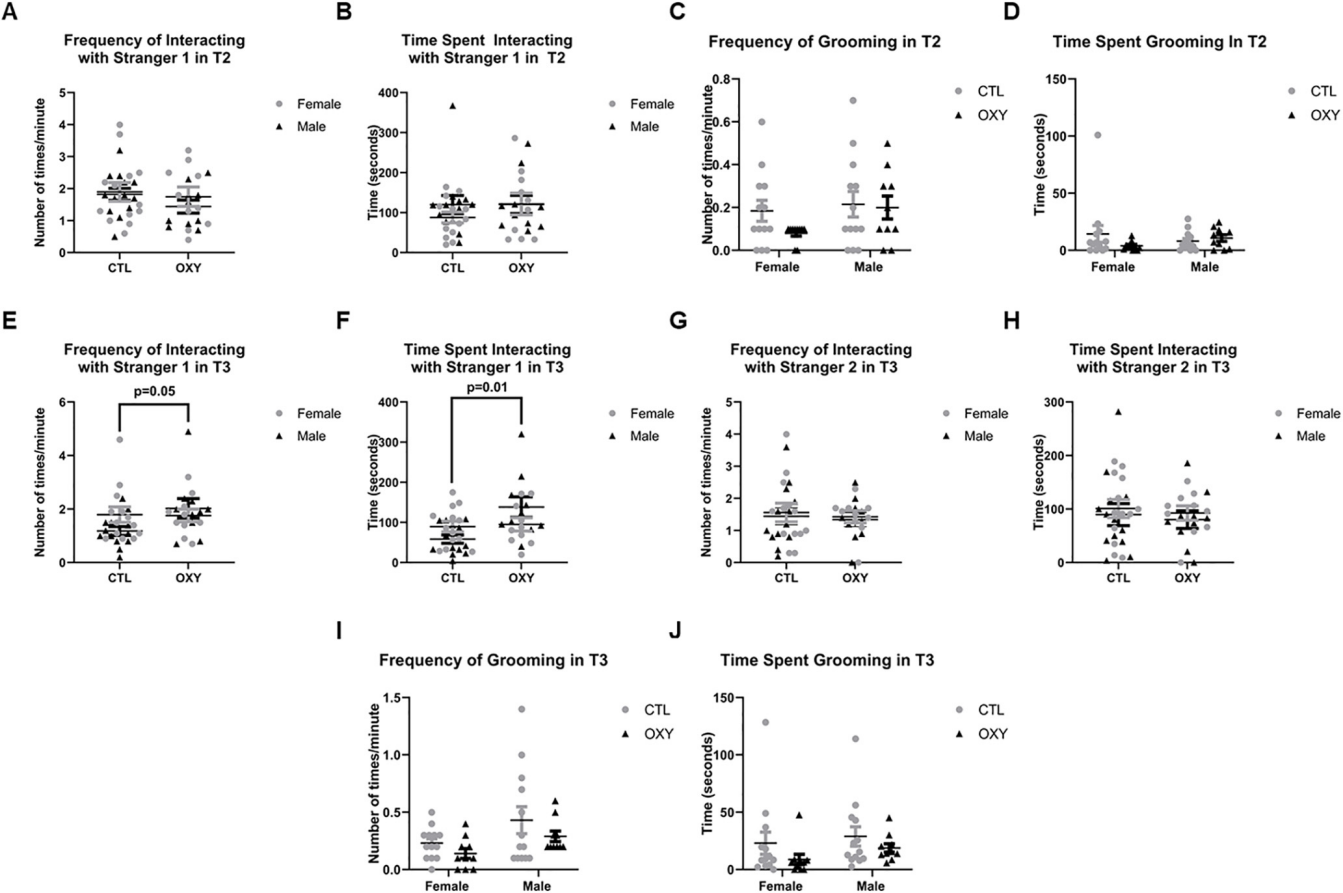
Social behavior results at adulthood. ***A***, Frequency of interacting with stranger 1 in T2. ***B***, Time spent interacting with stranger 1 in T2. ***C***, Frequency of grooming in T2. ***D***, Time spent grooming in T2. ***E***, Frequency of interacting with stranger 1 in T3. ***F***, Time spent interacting with stranger 1 in T3. ***G***, Frequency of interacting with stranger 2 in T3. ***H***, Time spent interacting with stranger 2 in T3. ***I***, Frequency of grooming in T3. ***J***, Time spent grooming in T3. The *F* test was used for data analysis. Treatment, sex, and treatment by sex interaction were included in the model. Treatment effects were analyzed with dam nested under treatment and used as the error term. There were significant (*p* ≤ 0.05) treatment effects on frequency and time spent in interacting with Stranger 1 in T3 as indicated in ***E***, ***F***. Error bars represent SEM. Number of replicates tested = 13 female and 13 male mice for CTL group, 10 female and 10 male mice for OXY group. Number of replicates tested = 13 female and 13 male mice for CTL group, 10 female and 10 male mice for OXY group.

**Figure 5.**
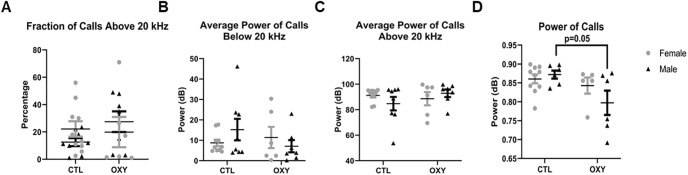
Audible and ultrasonic communications at adulthood. ***A***, Fraction of calls in the ultrasonic range (above 20 kHz). ***B***, Average power of calls below 20 kHz. ***C***, Average power of calls above 20 kHz. ***D***, Power of calls. The *F* test was used for data analysis. Treatment, sex, and treatment by sex interaction were included in the model. Treatment effects were analyzed with dam nested under treatment and used as error term. There was significant (*p* = 0.05) treatment effect on average power of calls as indicated in ***D***. Error bars represent SEM. Number of replicates tested = 13 female and 13 male mice for CTL group, 10 female and 10 male mice for OXY group. As with Figures 2 and 3, datapoints for all animals who engaged in these behaviors are shown. More animals called at this age than at PND21 to allow for inferences to be made, but some animals remained silent during the trial.

When social behaviors were tested again at 120 d of age, no differences in number of interactions or duration of time spent with stranger 1 mouse were noted in T2 (Fig. 4). The two groups also did not differ in grooming incidences or duration during T2 (Fig. 4). However, OXY-exposed offspring had increased incidences (df = 1, *F* = 4.1, *p* = 0.05) and duration (df = 1, *F* = 6.6, *p* = 0.01) investigating stranger 1 mouse in T3, suggestive that they preferred a familiar individual over a novel mouse (stranger 2; Fig. 4). No differences were noted in frequency and duration of time spent with stranger 2 mouse in T3. Grooming incidences and duration also did not differ in T3. For communication behaviors, the only difference noted at this age was that those exposed to OXY had reduced overall average power to their calls (Fig. 5). No differences in calls solely within the ultrasonic range or power of calls below and above 20 kHz were detected (Fig. 5). More animals called at this age than PND21 to allow for inferences to be made, but some animals remained silent during the trial.

### Barnes maze testing

**Figure 6.**
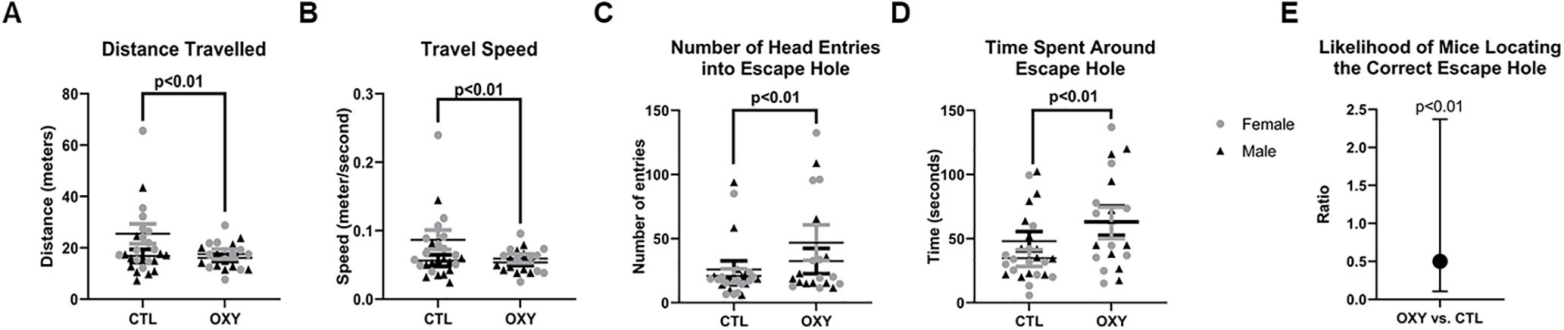
Barnes maze results. ***A***, Distance traveled. ***B***, Travel speed. ***C***, Number of head entries into escape hole. ***D***, Time spent around escape hole. ***E***, Likelihood of mice locating the correct escape hole. Distanced traveled, velocity, number of head entries into escape hole, and time spent around escape hole were analyzed as a split plot in space and time. Statistical model contained the fixed effects of treatment, sex, day, and all possible interactions with treatment, sex, and day. Latency was analyzed by using the PROC PHREG and proportional hazard ratio functions of SAS. A result <1 indicates that the treatment group is less likely to locate the correct escape hole compared with the CTL group. Number of replicates tested = 13 female and 13 male mice for CTL group, 10 female and 10 male mice for OXY group.

When tested in the Barnes maze, only treatment differences were noted. When male and female offspring were considered together, those exposed to OXY showed reduced distance traveled (df = 1, *F* = 21.8, *p* < 0.01) in this maze and reduced speed (df = 1, *F* = 24.8, *p* < 0.01; Fig. 6). Although OXY offspring were more likely to exhibit head entries around the escape hole and spent time around the escape hole, they less overall less likely to locate or actually go into the correct escape hole in the allotted 600 s relative to CTL as determined by using the hazard ratio approach (Fig. 6). This increased entries and time spent around the escape hole by OXY offspring could also thus reflect the fact that they were more likely to spend the entire 600-s duration in the maze, whereas their CTL counterparts found and went into the correct escape hole early on during the maze trial.

### EPM

**Figure 7.**
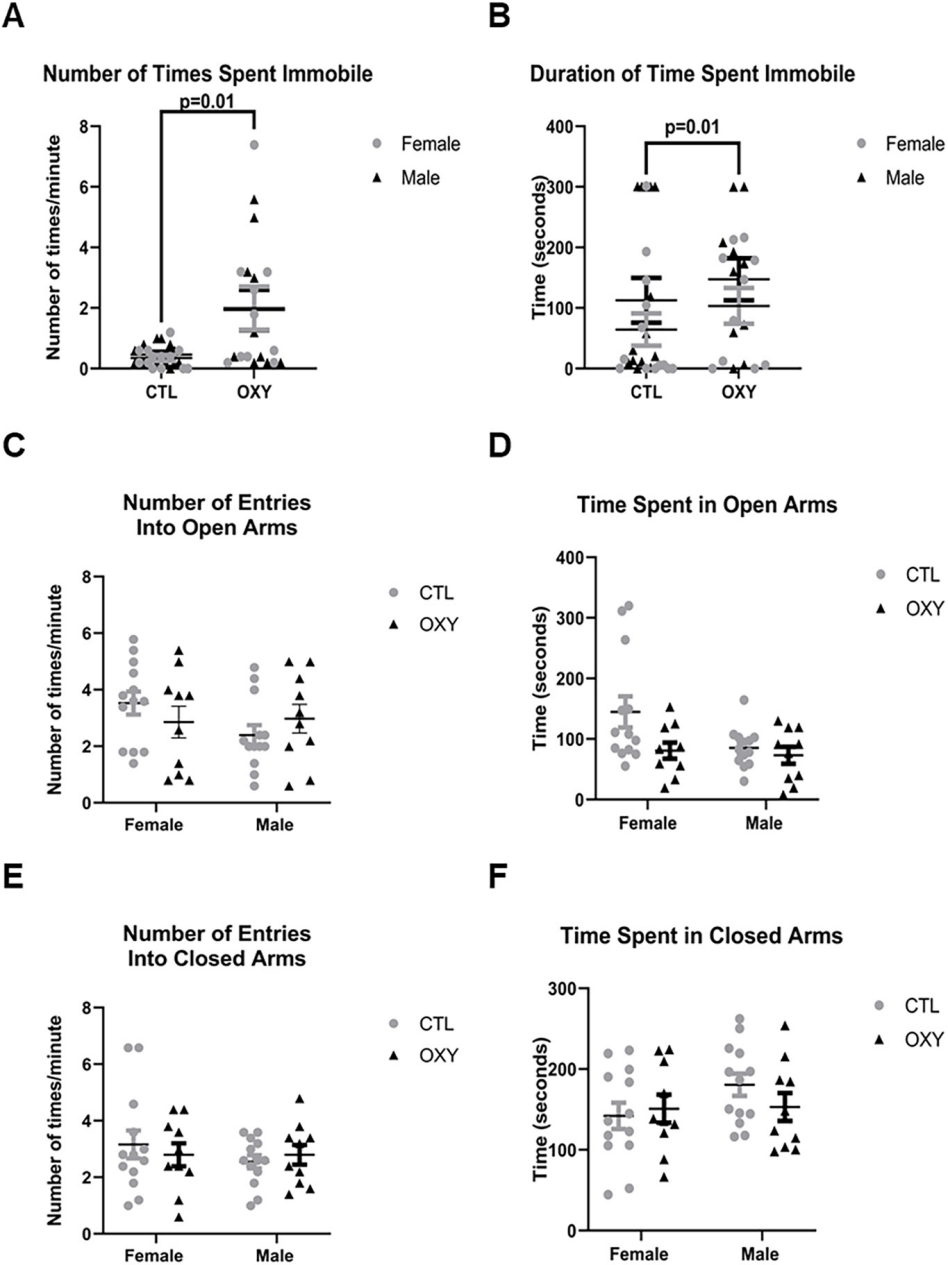
EPM results. ***A***, Number of times spent immobile. ***B***, Duration of time spent immobile. ***C***, Number of entries into open arms. ***D***, Time spent in open arms. ***E***, Number of entries into closed arms. ***F***, Time spent in closed arms. The *F* test was used for data analysis. Treatment, sex, and treatment by sex interaction were included in the model. Treatment effects were analyzed with dam nested under treatment and used as error term. There was significant (*p* = 0.01) treatment effect on number of times (***A***) and duration of time (***B***) spent immobile. Error bars represent SEM. Number of replicates tested = 13 female and 13 male mice for CTL group, 10 female and 10 male mice for OXY group.

During the EPM testing, male and female offspring showed increased frequency (df = 1, *F* = 6.9, *p* = 0.01) and duration (df = 1, *F* = 8.0, *p* = 0.01) of remaining immobile, suggestive of potential anxiety-like behaviors (Fig. 7). These findings are consistent with those observed in the Barnes maze of generally reduced voluntary physical activity. No differences, however, were noted in entries and duration of time spent in the open or closed arms of the maze (Fig. 7).

### Indirect calorimetry and echoMRI

**Figure 8.**
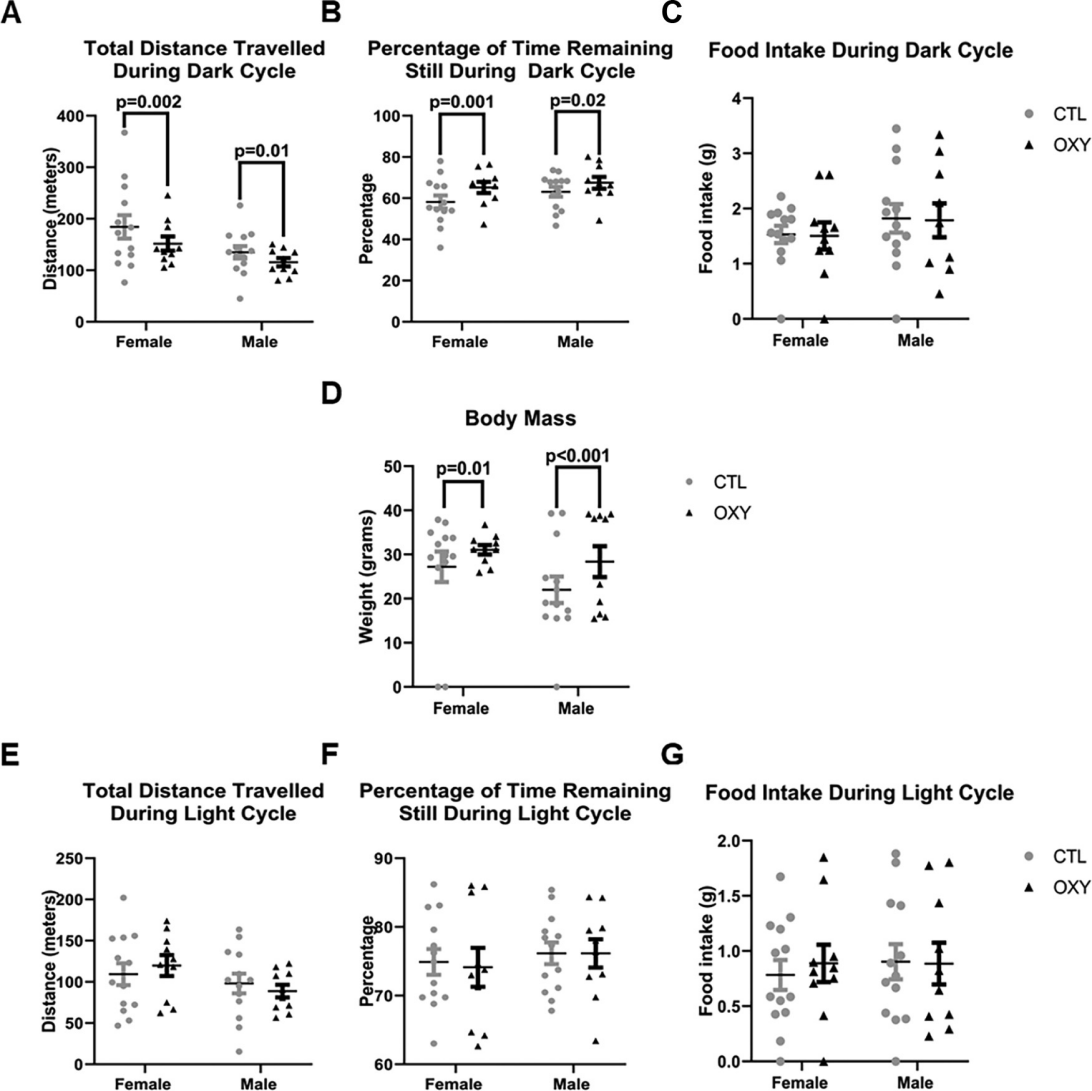
Indirect calorimetry and body mass results. ***A***, Total distance traveled during the dark cycle. ***B***, Percentage of time remaining still during the dark cycle. ***C***, Food intake during the dark cycle. ***D***, Body mass. ***E***, Total distance traveled during the light cycle. ***F***, Percentage of time remaining still during the light cycle. ***G***, Food intake during the light cycle. Data were analyzed with nested design and dam as experimental unit for treatment effects with treatment, sex, light cycle, and interactions between each of these factors as main effects. To illustrate possible sex differences, data are plotted separately for each sex. Significant differences are indicated in the figures. Number of replicates tested = 13 female and 13 male mice for CTL group, 10 female and 10 male mice for OXY group.

In this test, behaviors and other assessments are divided into dark and light cycles with the dark cycle considered the most active period for rodents. However, male and female offspring exposed to OXY showed reduced distance traveled during this period (Fig. 8). Correspondingly, both OXY male and female offspring were more likely to remain still during the dark cycle relative to controls (Fig. 8). Presumably because of this reduced voluntary physical activity, both male and female offspring exposed to OXY had an increase in body mass (Fig. 8). However, no differences in fat content were detected with echoMRI. No differences in general activity were noted during the light cycle when the mice generally sleep (Fig. 8). Food intake did not differ for the two groups during the dark or light cycle (Fig. 8).

### qPCR

**Figure 9.**
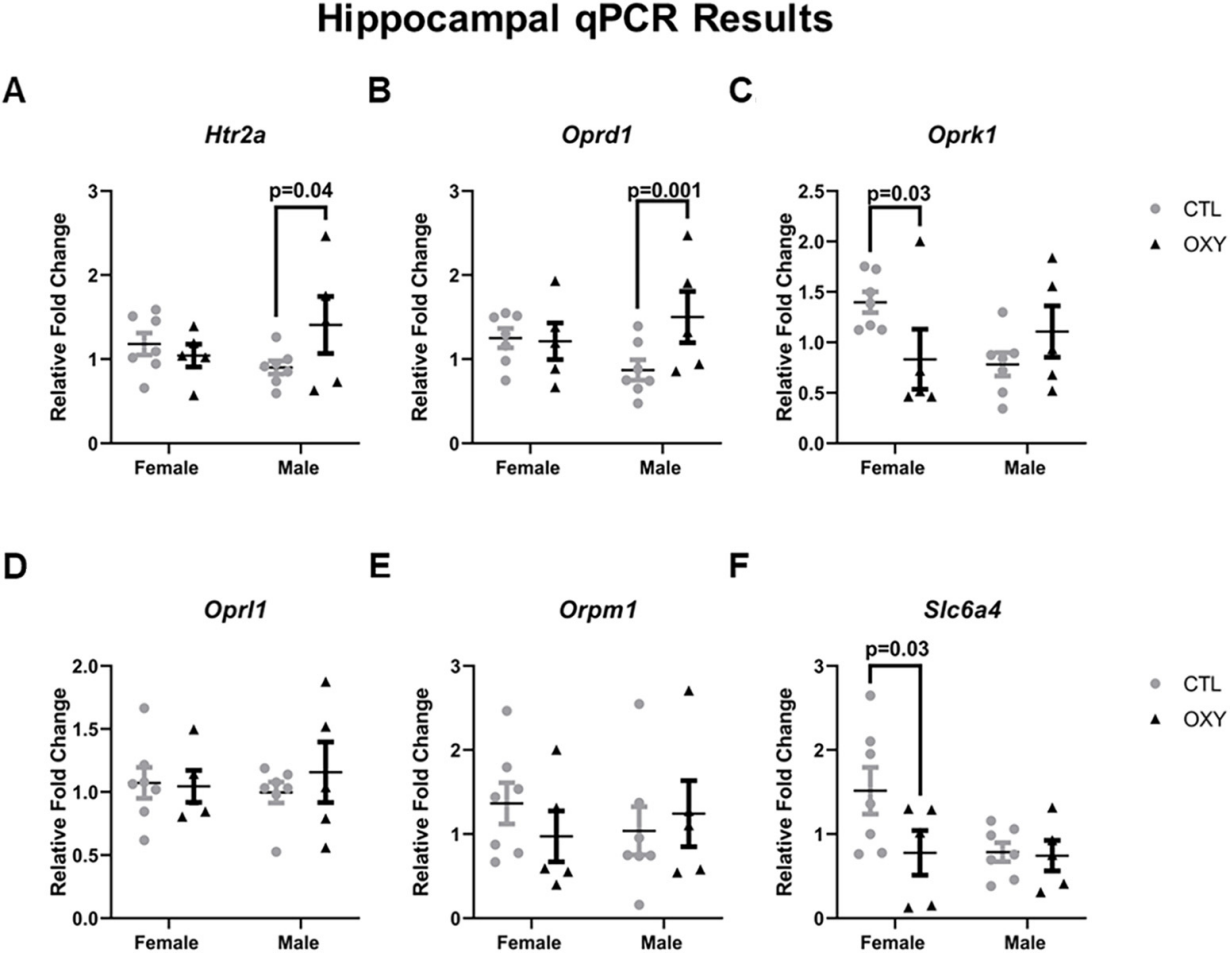
Gene expression in hippocampal tissue. Gene expression data, as determined by qPCR assay, were normalized by using combined average dCt values of the two housekeeping genes: *B2m* and *Rpl7* and then analyzed based on treatment, sex, and their interactions, and dam was considered the experimental unit for treatment effects. Significant differences are indicated in the figures. Gene expression patterns for: ***A***) Htr2a; ***B***) Oprd1; ***C***) Oprk1; ***D***) Oprl1; ***E***) Oprm1; ***F***) Slc6a4. Number of replicates tested = seven female and seven male mice for CTL group, five female and five male mice for OXY group. Additional qPCR hippocampal results are included in Extended Data Figure 9-1.

Of the genes tested, four showed treatment × sex differences in the hippocampus (Fig. 9). Included in this figure are the results for the opioid receptors to which OXY might bind, *Oprm1* and *Oprl1*, but no treatment or treatment × sex interaction affects were noted for these two genes. For *Oprd1*, male offspring exposed to OXY showed increased expression of this gene in the hippocampus. In contrast, *Oprk1* had reduced expression in the hippocampus of females exposed to OXY. The 5-HT receptor, *Htr2a*, was increased in male hippocampi exposed to OXY. However, the gene encoding the 5-HT transporter (SERT), *Slc6a4*, was reduced in female hippocampi exposed to OXY. No other gene expression differences were observed for those examined (Fig. 9; Extended Data Fig. 9-1)

10.1523/ENEURO.0150-21.2021.f9-1 Extended Data Figure 9-1 Additional gene expression in hippocampal tissue not included in Figure 9. Gene expression data, as determined by qPCR assay, were normalized by using combined average dCt values of the two housekeeping genes: *B2m* and *Rpl7* and then analyzed based on treatment, sex, and their interactions, and dam was considered the experimental unit for treatment effects. Number of replicates tested = seven female and seven male mice for CTL group, five female and five male mice for OXY group. Download Figure 9-1, TIF file.

**Figure 10.**
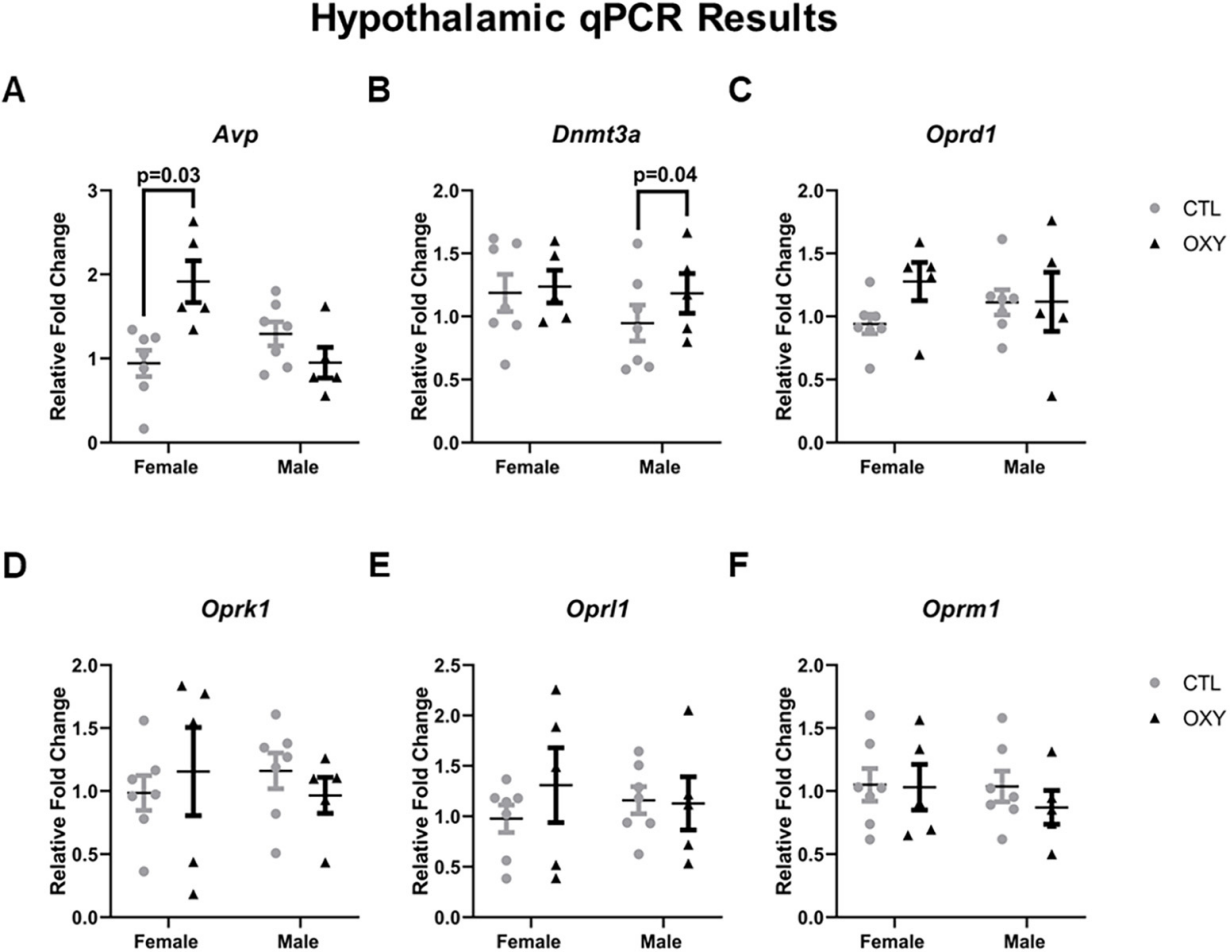
Gene expression in hypothalamic tissue. Gene expression data, as determined by qPCR assay, were normalized by using combined average dCt values of the two housekeeping genes: *B2m* and *Rpl7* and then analyzed based on treatment, sex, and their interactions, and dam was the considered experimental unit for treatment effects. Significant differences are indicated in the figures. Gene expression patterns for: ***A***) Avp; ***B***) Dnmt3a; ***C***) Oprd1; ***D***) Oprk1; ***E***) Oprl1; ***F***) Oprm1. Number of replicates tested = seven female and seven male mice for CTL group, five female and five male mice for OXY group. Additional qPCR hippocampal results are included in Extended Data Figure 10-1.

None of the opioid receptors showed expression differences related to OXY exposure or the interaction of OXY exposure and offspring sex (Fig. 10). *Avp* was increased in the hypothalamus of females exposed to OXY. *Dnmt3a* had increased relative expression in the hypothalamus of male offspring exposed to OXY (Fig. 10). No other gene expression differences were detected for those surveyed (Fig. 10; Extended Data Fig. 10-1).

10.1523/ENEURO.0150-21.2021.f10-1 Extended Data Figure 10-1 Additional gene expression in hypothalamic tissue not included in Figure 10. Gene expression data, as determined by qPCR assay, were normalized by using combined average dCt values of the two housekeeping genes: *B2m* and *Rpl7* and then analyzed based on treatment, sex, and their interactions, and dam was the considered experimental unit for treatment effects. Number of replicates tested = seven female and seven male mice for CTL group, five female and five male mice for OXY group. Download Figure 10-1, TIF file.

### Integrative correlation analyses

**Figure 11.**
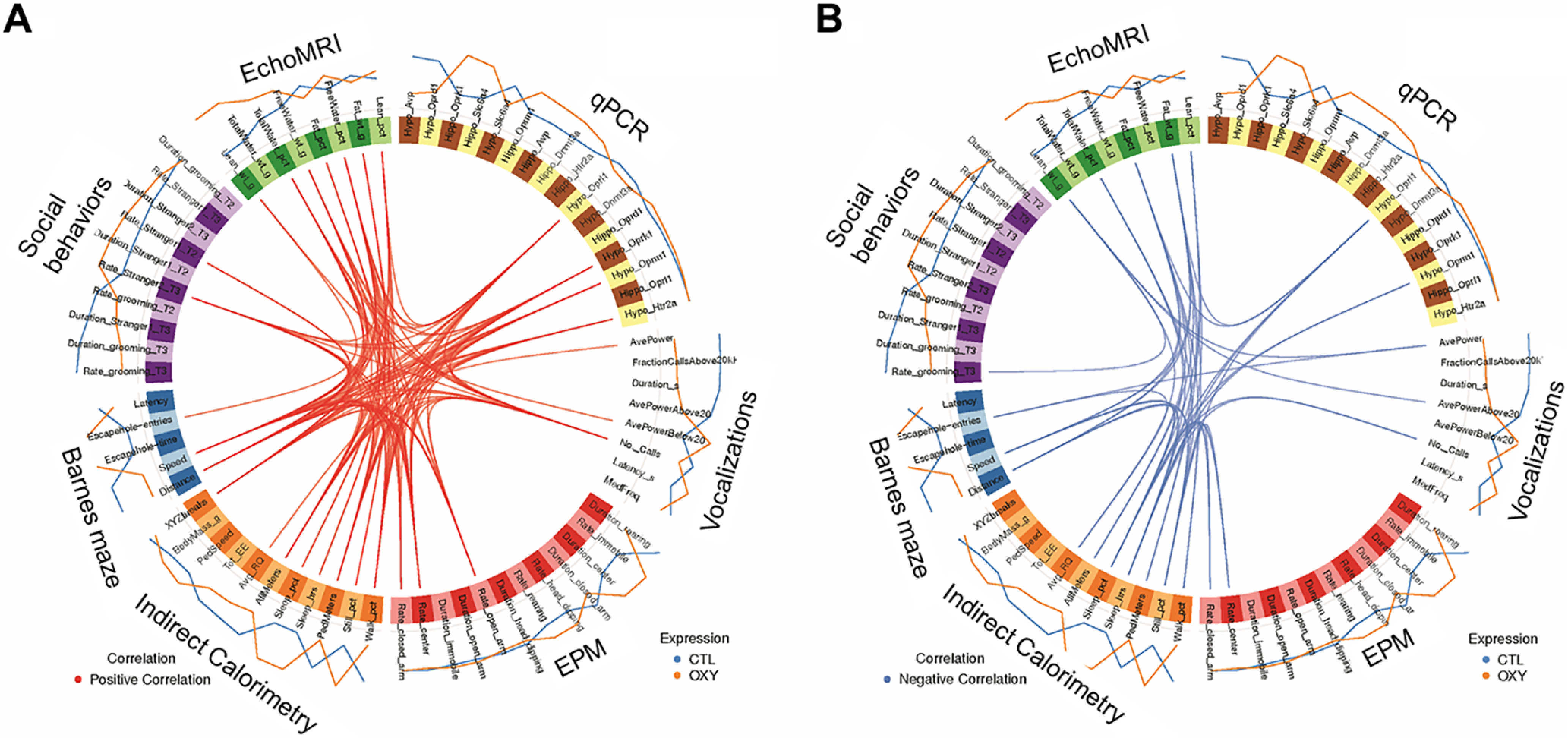
Circos plot correlations for OXY-exposed versus CTL female mice. mixOmics R program (v6.1.1) was used to correlate Barnes maze, social, EPM, indirect calorimetry, echoMRI, and gene expression data. Correlation cutoff was set to 0.75. Red lines in the center indicate a positive correlation (***A***), whereas blue lines (***B***) indicate a negative correlation. Lines on the outside of the circle indicate whether the value was greater in CTL (blue) versus OXY (orange).

MixOmics analyses with a ≥0.75 correlation value, which is considered quite stringent, revealed several positive and negative correlations in both female and male groups. In females, hypothalamic expression of *Oprk1*, *Oprl1*, *Oprm1*, and *Htr2a* positively associated with number of calls, speed and distance traveled in the Barnes maze, and all meters, ped meters, and walking percentage during indirect calorimetry testing (Fig. 11). In contrast, *Oprl1* expression in females inversely associated fat weight and fat percentage as measured by echoMRI and sleep hours, sleep percentage, and still percentage for indirect calorimetry testing (Fig. 11). *Oprk1* expression in females was inversely associated with sleep hours, and *Oprm1* negatively correlated with sleep hours, and still percentage.

**Figure 12.**
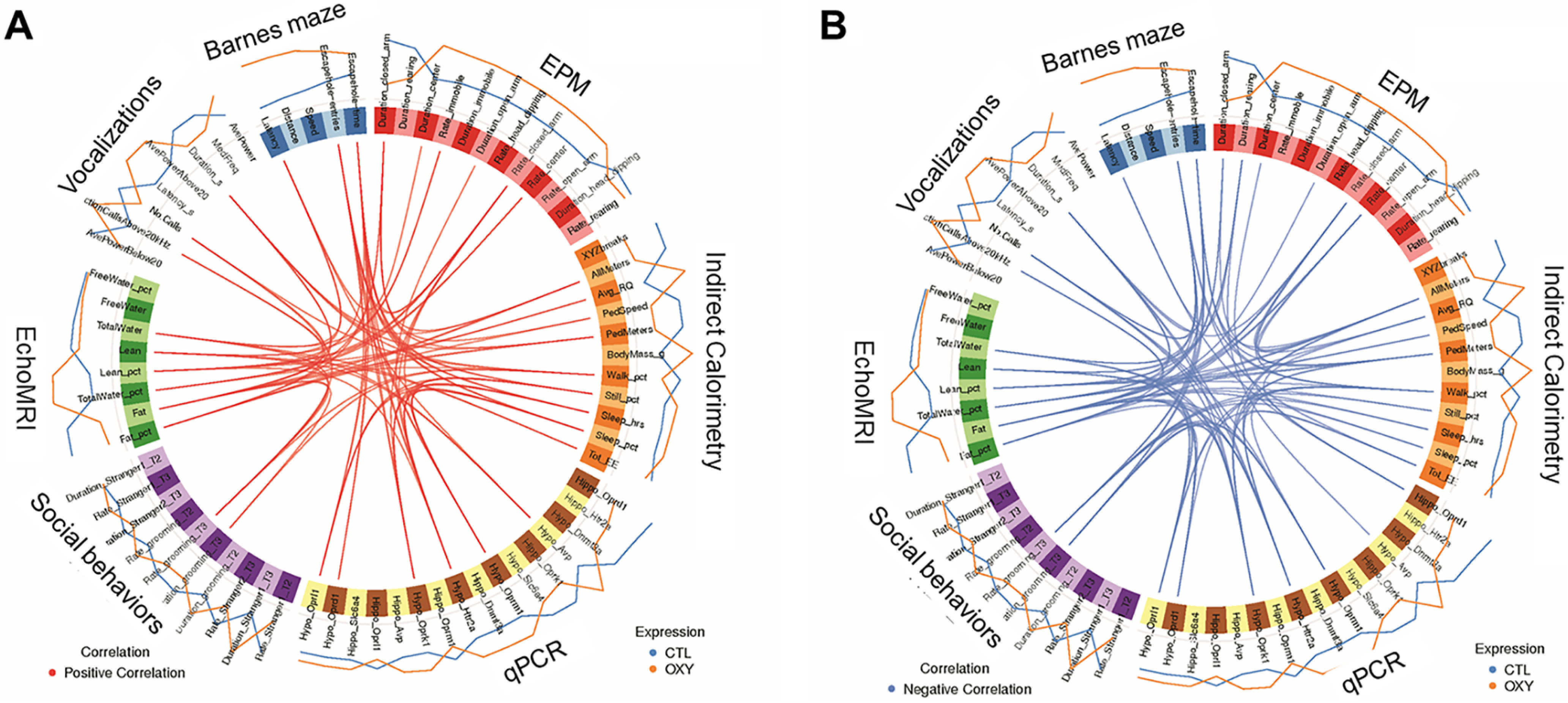
Circos plot correlations for OXY-exposed versus CTL groups of male mice. mixOmics R program (v6.1.1) was used to correlate Barnes maze, social, EPM, indirect calorimetry, echoMRI, and qPCR testing. Correlation cutoff was set to 0.75. Red lines in the center indicate a positive correlation (***A***), whereas blue lines (***B***) indicate a negative correlation. Lines on the outside of the circle indicate whether the value was greater in CTL (blue) versus OXY (orange).

In males, *Oprd1*, *Oprl1*, *Oprm1*, and *Htr2a* positively associated with average power of calls (Fig. 12). Hypothalamic expression of *Oprk1* also positively correlated with still percentage, sleep percentage, and sleep hours, which is the opposite linkage detected in females. *Oprk1*, *Oprm1*, and *Htr2a* were positively linked with speed and distance traveled in the Barnes maze. Hypothalamic expression of *Avp* positively correlated with total body water, which is consistent with the known role as an anti-diuretic. In males, *Oprl1* was inversely associated with total water, whereas Oprd1 negatively correlated with number of calls (Fig. 12). *Oprk1*, *Oprm1*, and *Htr2a* was inversely linked to duration of calls, rate of grooming during social testing, and all meters and ped meters traveled in indirect calorimetry testing.

## Discussion

The goals of the current studies were to determine the effects of maternal exposure to OXY on mouse offspring socio-communication behaviors at weaning (PND21) and adulthood. At adulthood, additional behaviors and metabolic parameters that were examined included cognition, anxiogenic and exploratory behaviors, voluntary physical activity, and body weight. At weaning and adulthood, there was evidence of reduced social behaviors in OXY-exposed offspring as evidenced by decreased frequency and duration interacting with the stranger mouse in T2 in the social behavior testing for weanling mice. However, such effects did not carry over to the T3 for weanling mice with no differences observed in terms of frequency or time spent with stranger 1 or 2 mice. At adulthood, OXY-exposed mice spent more time engaging with the first stranger mouse than the second stranger mouse in T3. No differences though were noted in T2 at this age. Collectively, there are some suggestions of impaired social behaviors in those exposed to OXY, but the effects seem to vary with age and are not fully consistent across trials. For communication in the audible and ultrasonic range, age differences were also noted. While weanling (PND30) mice exposed to OXY were more likely to communicate in the ultrasonic range, the power of their calls was reduced at adulthood. As rodent pups tend to communicate with parents in the ultrasonic range (Rosenfeld et al., 2013), it could be that OXY-exposed individuals are more likely to persist engaging in such calls through weaning. Further work examining neonatal pups up through adults may be useful in sorting out these effects and potential explanations.

In offspring born to Sprague Dawley rats allowed to self-administer OXY, increased OXY intake by the dams is associated with fewer calls at PND3, but this pattern shifts at PND9 with higher OXY intake linked with greater number of calls (Vassoler et al., 2018). However, another study with rats showed that that in utero exposure to 0.5 mg/kg/d OXY did not affect USVs produced in isolation during the early postnatal period or learning and memory in at adulthood (Sithisarn et al., 2017). However, these rats were hyperactive at adulthood. In other work, in utero and postnatal exposure of Sprague Dawley rats to OXY results in enhanced anxiety-like behaviors and social deficits that is transmitted to subsequent generations (Odegaard et al., 2020). This study also revealed that OXY exposure during these time periods or in F2 offspring resulted in gene expression changes in the nucleus accumbens (NAc) for key synaptic genes and neuropeptides involved with the hypocretin system, which has a role in addiction. Prenatal exposure of Sprague Dawley rats to other opioid drugs, methadone or buprenorphine, resulted in later social deficits and impaired cognition as evidenced by performance in the novel object recognition test (Chen et al., 2015).

In the current study, measurements of general activity and spatial learning and memory at adulthood in the Barnes maze revealed that OXY-exposed offspring generally traveled less distance, were slower, and less likely to find the escape hole relative to CTL. The findings thus suggest that prenatal exposure to OXY can compromise spatial learning and memory and/or motivation to locate the correct escape hole. While the aforementioned rat study did not find any evidence that early exposure to OXY affects later cognition (Sithisarn et al., 2017), other reports support the current findings. Male offspring born to female Sprague Dawley rats treated for 30 d via oral gavage with OXY (incremental concentration achieving 15 mg/kg/d) throughout breeding and gestation, demonstrated impairments in spatial learning and memory when tested in the T-maze and Morris water maze (Davis et al., 2010). Spatial memory of male but not female Wistar rats and grand-offspring is compromised in those derived from dams treated with morphine (Odegaard et al., 2020). *Mecp2* and *Hdac2* were significantly upregulated in the hippocampus of these male groups. Rat offspring derived from female and male parents treated with morphine exhibit memory deficiency that is presumably because of suppression of long-term potentiation in the hippocampus (Sarkaki et al., 2008). Paternal exposure of Sprague Dawley rats to morphine impairs novel-object recognition in female but not male progeny (Ellis et al., 2020).

When OXY and CTL mice were tested in the EPM, they were more likely to remain immobile during this test. However, no differences in entries or time spent in the open or closed arms was evident. Thus, the findings might suggest that the mice had reduced motivation to move around rather than anxiogenic behaviors. If so, such results would be consistent with the general lack of movement in the Barnes maze. Furthermore, indirect calorimetry studies revealed OXY-exposed individuals showed reduced voluntary physical activity during the dark cycle, which is considered the height of general activity. Additional tests that measure motivation would be needed to confirm the potential effect of OXY on this behavioral trait. A previous study with rats reported that developmental exposure to morphine increased anxiety-like behaviors, but such effects were mitigated by stimulating IGF-2 signaling and providing environmental enrichment (Li et al., 2014).

Here, we noted that grooming behavior, which may be considered a stereotypic behavior, was not consistently affected across the trials during the social behavior testing. A study with rats found that males derived from pairings where one or both parents was treated with morphine exhibited increased grooming behavior (Rohbani et al., 2019). Marble burying behavior was also increased in such groups, collectively suggesting that such early exposure may increase obsessive compulsive-like behaviors. The contrasting findings might be attributed to animal model, mouse versus rat. Additionally, it is clear that different opioids can exert differential effects through activation of varying opioid receptors. Thus, we also examined opioid receptors and other associated genes in the hippocampus and hypothalamus.

Our prediction was that prenatal exposure to OXY would induce longstanding changes on gene expression for its own receptors, especially *Oprm1*, in the hippocampus and hypothalamus. These brain regions were examined because of their role in regulating learning and memory and social behaviors, respectively. While OXY exposure did not affect expression for *Oprm1* in either brain region, this opioid induced brain-specific and sex-specific differences for other opioid receptors. In the hippocampus, *Oprd1* was elevated in OXY-exposed males, whereas *Oprk1* had reduced expression. In the same Sprague Dawley rat study that showed that maternal self-administration of OXY associated with calls emitted at the various PND ages, the investigators also found that maternal OXY exposure to did not affect *Oprm1* expression in the hypothalamus or forebrain region at PND1 (Vassoler et al., 2018). However, maternal OXY exposure dramatically decreased expression of this opioid receptor form in the midbrain region at this age. Injection of buprenorphine (2.5 mg/kg) into dams increases kappa1 opioid receptors, as detected with the kappa1-selective agonist 3H-U69593, in the brain of PND1 rats (Belcheva et al., 1998). However, this same treatment downregulates neural μ-opioid receptor density.

Aspects of 5-HT signaling in the hippocampus were also likely disrupted because of OXY exposure with *Htr2a* increased in male hippocampi, but *Slc6a4* exhibiting reduced expression in OXY-exposed female hippocampi. The results indicate that OXY-exposed males might have more 5-HT receptors to bind to 5-HT. Reductions in SERT in their female siblings may result in less internalization of 5-HT and thereby more of this neurotransmitter available to bind and activates its receptor. Thus, the net effect in both males and females exposed to OXY might be enhanced 5-HT signaling in the hippocampus.

In the hypothalamus, none of the opioid receptors differed based on OXY exposure. Increase expression of *Avp*, which often counteracts oxytocin, may account for reductions in social behaviors because of OXY exposure. Increased expression of *Dnmt3a* in OXY males may lead to hypermethylation of select genes. Follow-up studies will thus examine DNA methylation patterns and other epigenetic changes in these two brain regions. One brain region, we did not examine in the current studies but that will be the focus of future work is the nucleus accumbens (NAc) region. It is the primary brain region guiding motivation to engage in voluntary physical activity (Odegaard et al., 2020). As mentioned previously, OXY exposure of rats during the prenatal or postnatal period altered synaptic genes and neuropeptides involved with the hypocretin system, in this brain region, and such effects were observed in F2 offspring who were not directly exposed to the drug (Odegaard et al., 2020). It will thus be of interest to see whether similar gene expression differences are observed in this brain region in mice exposed to OXY.

Integrative correlation analyses revealed strong correlations between expression of hypothalamic expression of opioid receptors, including *Oprd1*, *Oprk1*, *Oprl1*, and *Oprm1*, and *Htr2a* and general activity when tested in the indirect calorimetry unit, fat content, activity in the Barnes maze, and vocalizations. However, for some correlations the directionality differed based on female versus male offspring with hypothalamic expression of *Oprd1*, *Oprk1*, *Oprl1*, and *Oprm1*, and *Htr2a* positively correlating for increased activity level in females. In contrast, these genes were negatively associated with increased activity levels in males. None of the hypothalamic genes for the opioid receptor forms differed based on OXY exposure, and thus, the relevance of these relationships remains uncertain. Interestingly, maternal obesity during the periconception or gestational period increased expression of *Oprm1* in the NAc of male offspring when measured at 14 weeks of age, whereas, maternal periconceptional obesity decreased expression of *Oprm1* in the NAc of female siblings (Grissom et al., 2014). Such findings lend further support for a relationship between neural expression of opioid receptors, activity, and adiposity that merits further examination. Follow-up transcriptomic studies are underway to examine how developmental exposure to OXY affects global expression patterns in the hypothalamus, NAc, and hippocampus.

In conclusion, developmental exposure to OXY may disrupt aspects of social behaviors that persist through adulthood. Such offspring also showed cognitive impairments and reduced voluntary physical activity. OXY-exposed offspring weighed more than CTL counterparts, assumingly because of reductions in spontaneous physical activity. In the hippocampus, prenatal exposure to OXY caused sex-dependent differences in expression of genes encoding opioid receptors and those involved in 5-HT signaling. OXY exposure induced changes in neuropeptide hormone expression and the epigenetic modulator, *Dnmt3a*, in the hypothalamus. Follow-up studies will determine the epigenetic changes induced in the brain by early OXY exposure. The findings though suggest cause for concern that consumption of OXY by expecting mothers may result in permanent neurobehavioral changes in their offspring.

## Synthesis

Reviewing Editor: Leandro Vendruscolo, The Scripps Research Institute Decisions are customarily a result of the Reviewing Editor and the peer reviewers coming together and discussing their recommendations until a consensus is reached. When revisions are invited, a fact-based synthesis statement explaining their decision and outlining what is needed to prepare a revision will be listed below. The following reviewer(s) agreed to reveal their identity: Khaled Moussawi, Renata Marchette.

Reviewer 1

The authors aimed to study the long lasting effects of maternal opioid exposure on different cognitive and behavioral domains in offspring mice: learning and memory, anxiety, social interaction, vocalization, etc...

As the authors mention in the introduction and discussion sections, there has been many studies evaluating the effects of maternal oxycodone and other opioids exposure on these different domains in rodent offsprings (mostly rats). Some of the results in this study support while others contradict published studies.

This study seems more comprehensive than prior studies; however, key data from the experiments performed by the authors were not presented and many conclusions are not well supported by the data. Also, the statistical analyses are not vey clear.

The authors provide a general overview of statistical analyses in the methods section. However, no statistical details are provided in the results or figure legends. It is not clear to me what tests and analyses were used for each figure/result.

This study complements a body of literature regarding the effects of prenatal exposure to opioids on neurodevelopment. Some suggestions to the authors:

- it would be useful to include the individual data points in the figures for both males and females instead of just presenting histograms. males and females can be differentiated using different colors or symbols.

- it would be useful to include the specific statistical tests and F scores/df for the different results.

- It is not clear why the authors included some but not other data in their results/figures. For example: For the social interaction experiment, Figure 1 (PND21) shows social interaction in T2 and grooming in T3. The figure does not show grooming for T2 and social interaction for T3. On the other hand, for the same experiment at PND 120 (Figure 3), social interaction is shown for T3. As presented, the data presentation appears selective of what seems to support the author's hypothesis. We recommend that all data are presented and discrepancies reconciled.

- Similarly, Figure 2 shows vocalization data >20KHz. It is not clear how the overall vocalization data at all frequencies compare between groups. And what is the significance of the potential selective effect on ultrasonic vocalizations vs. audible frequencies?

- the authors make some strong statements about motivation being affected (based on locomotor data in Barnes test and EPM). However, these would have been better supported with dedicated tasks to test for motivation.

- Also, the conclusions about anxioigenic behaviors are not fully supported. The data regarding open vs. closed arms entries in the EPM test are not presented (the discussion mentioned these were not different). The only results shown in this task were time of immobility. These results do not support a conclusion of increased anxiety like behaviors.

- what did the calorimetric tests show during the light cycle?

- how were the statistics done for the qPCR data? were these corrected for multiple comparisons for the genes tested? the male oprd1 results in Figure 7 were especially curious.

- why were the tested genes selected differently between the hippocampus vs hypothalamus?

Reviewer 2

The manuscript presents data showing lasting behavioral and metabolic effects of in uterus exposure to opioids that the author correlate to gene expression in the hippocampus and hypothalamus. Giving the high incidence of OUD, and its growing rate in women, a better understanding of the consequences of pre-natal opioid exposure is highly important.

The authors did not report the statistics in the results description.

In their manuscript 'Maternal oxycodone treatment results in neurobehavioral disruptions in mice offspring' the authors present a extensive behavioral and physiological analysis, as well as gene regulation in the hippocampus and hypothalamus, in offspring from mother exposed to oxycodone or vehicle. they report alterations in social interaction, ultrasonic vocalizations and spatial memory. The manuscript is well design and I believe these results will significantly advance the field, but I believe there some points that need to be addressed before it is suitable for publication.

1. what is the light cycle in which the animals are kept?

2. Are there litter size differences or differences in the female/male ration between control and OXY? I assume the authors used only one mouse of each sex per litter due to variabilities in these ratios. Would be beneficial to have these information stated.

3. The measurements and illumination level of the mazes is missing.

4. A timeline of the whole experiment would facilitate the contextualization of the data.

5.At what bregma level were the punches taken? Did they comprised only dorsal hippocampus? The authors state that the punches spanned from rostral to caudal medial regions but it is not clear to which brain region they are referring to.

6. Please include all the statistical analysis values throughout the results section. In occasions where males and females are grouped together, after no sex differences where identified, it would be greatly beneficial if they showed the individual data points as male and female symbols.

7. What's the significance of grooming behavior during social interaction test?

8. If the animals have impaired locomotion, as it is shown on both the Barnes maze and the spontaneous locomotion assessment, it is not possible to infer an anxiogenic-like effect in the EPM. Since this is a confounding factor in this test. Please show open arms exploration. Where do the immobility episodes occur? Also, based on the tracings in figure 10, looks like OXY males might spend more time in the open arms than CTL and engage in more head dipping, which could indicate an increase in risk-taking behavior.

9.Why no measures of spatial memory/learning from the Barnes maze where included in the correlation analysis?

10. The authors should considering including all the behavioral and metabolic measures analyzed as supplemental table or figures.

11. What was the reasoning behind not analyzing all mice for gene expression? And that should have been stated in the methods, not only in the figure legends.

Do the groups differ on food intake or only on general activity?

12. The reference style should be standardized throughout the manuscript.

13. Careful proof reading and copy editing is necessary.
