# Supplementary figures and images for: Maternal Oxycodone Treatment Results in Neurobehavioral Disruptions in Mice Offspring
Source: eNeuro. 2021 Aug 4;8(4):ENEURO.0150-21.2021. doi: 10.1523/ENEURO.0150-21.2021 (PMC8354714; doi:10.1523/ENEURO.0150-21.2021)

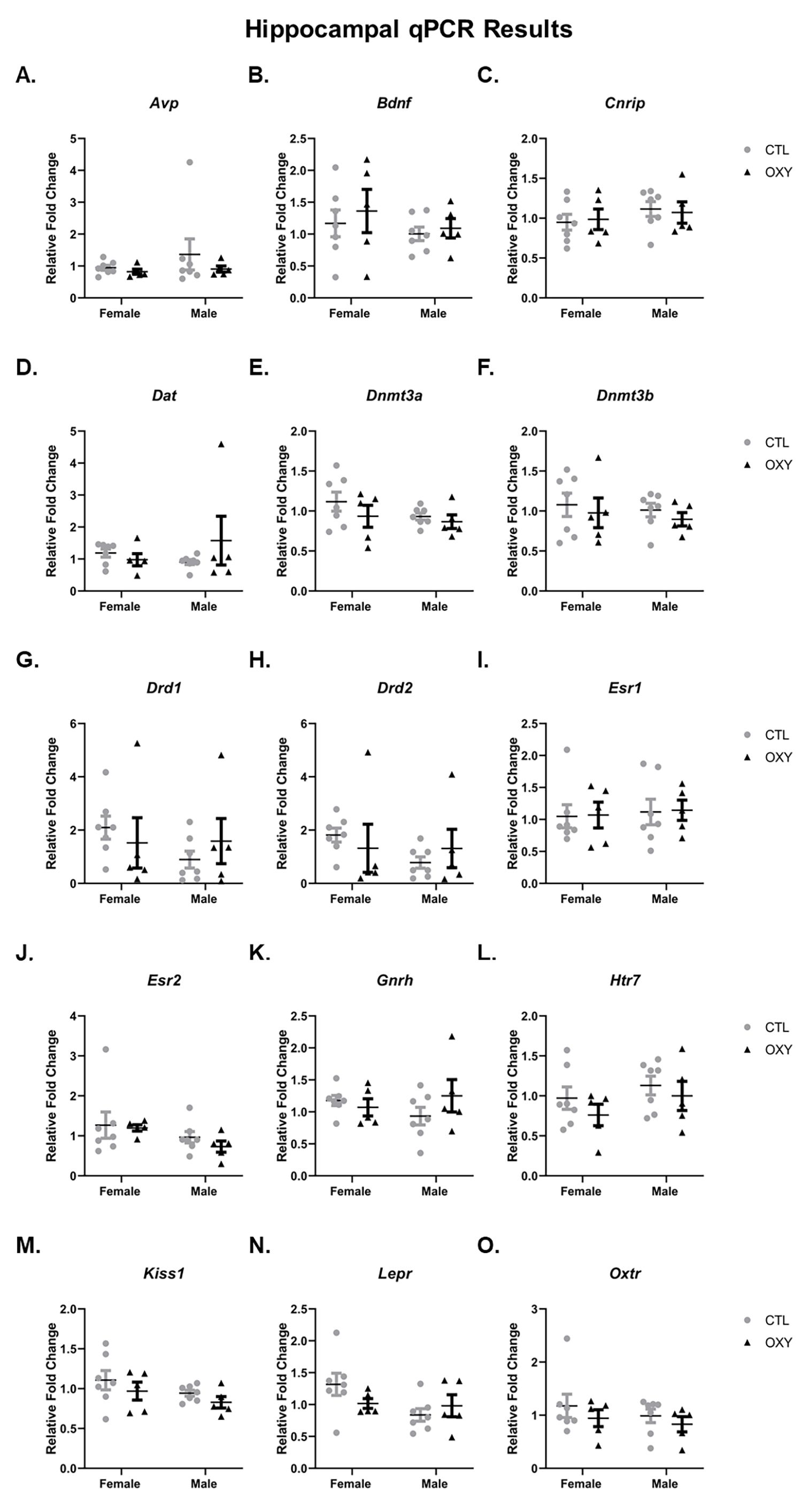

Supplement: Extended Data Figure 9-1 — Additional gene expression in hippocampal tissue not included in Figure 9. Gene expression data, as determined by qPCR assay, were normalized by using combined average dCt values of the two housekeeping genes: B2m and Rpl7 and then analyzed based on treatment, sex, and their interactions, and dam was considered the experimental unit for treatment effects. Number of replicates tested = seven female and seven male mice for CTL group, five female and five male mice for OXY group. Download Figure 9-1, TIF file. [file enu-eN-NWR-0150-21-s02.tif]

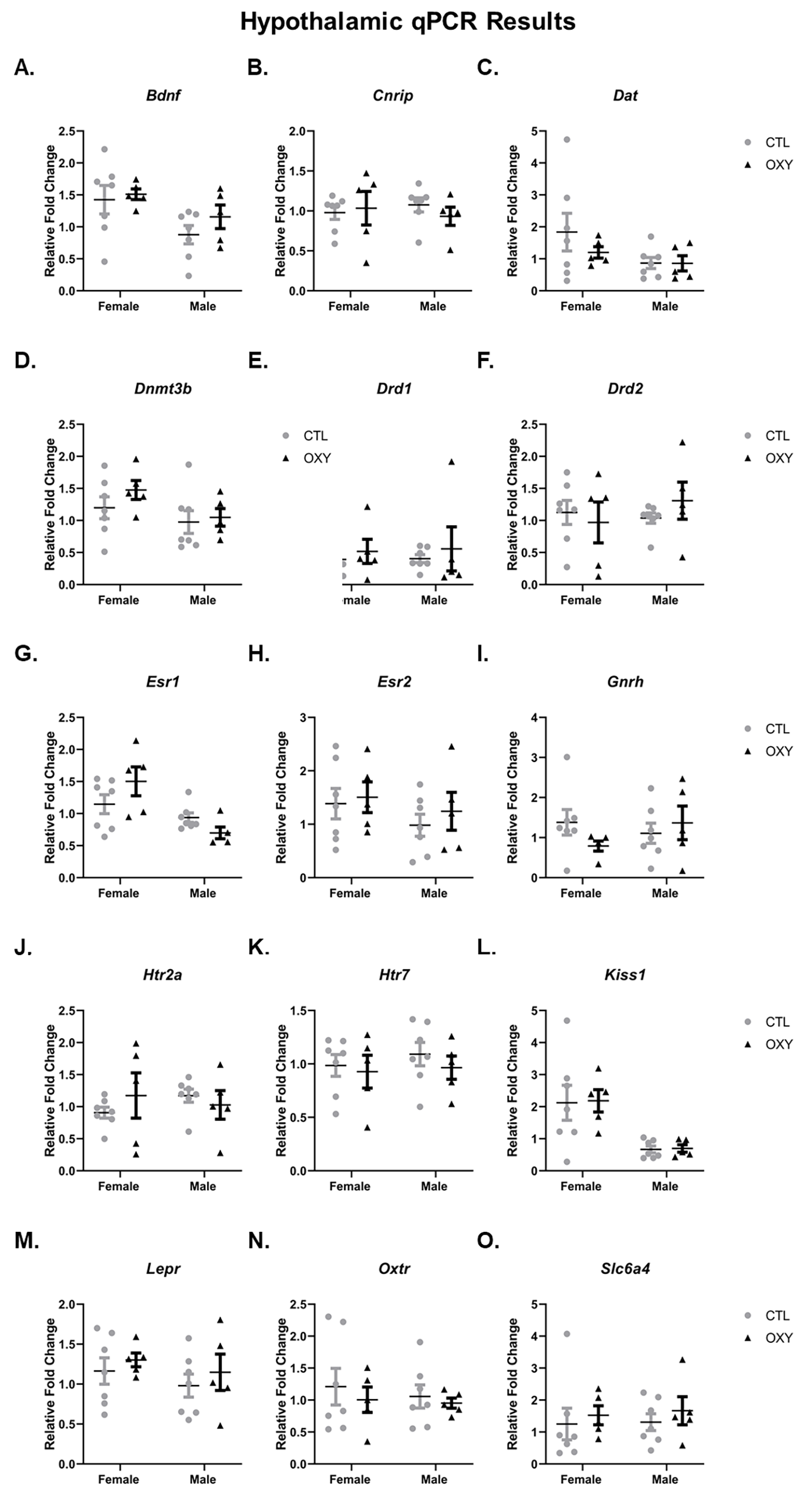

Supplement: Extended Data Figure 10-1 — Additional gene expression in hypothalamic tissue not included in Figure 10. Gene expression data, as determined by qPCR assay, were normalized by using combined average dCt values of the two housekeeping genes: B2m and Rpl7 and then analyzed based on treatment, sex, and their interactions, and dam was the considered experimental unit for treatment effects. Number of replicates tested = seven female and seven male mice for CTL group, five female and five male mice for OXY group. Download Figure 10-1, TIF file. [file enu-eN-NWR-0150-21-s03.tif]
